# Diphenyl diselenide protects neuronal cells against oxidative stress and mitochondrial dysfunction: Involvement of the glutathione-dependent antioxidant system

**DOI:** 10.1016/j.redox.2018.09.014

**Published:** 2018-09-25

**Authors:** Ruth Liliám Quispe, Michael Lorenz Jaramillo, Leticia Selinger Galant, Daiane Engel, Alcir Luiz Dafre, João Batista Teixeira da Rocha, Rafael Radi, Marcelo Farina, Andreza Fabro de Bem

**Affiliations:** a Neuroscience PhD Program, Department of Biochemistry, Federal University of Santa Catarina, Florianópolis, SC, Brazil; b Department of Cell Biology, Embryology and Genetics, Federal University of Santa Catarina, SC, Brazil; c Biochemistry PhD Program, Department of Biochemistry, Federal University of Santa Catarina, Florianópolis, SC, Brazil; d Department of Biochemistry and Molecular Biology, Federal University of Santa Maria, Santa Maria, RS, Brazil; e Department of Biochemistry and Center for Free Radical and Biomedical Research (CEINBIO), Facultad de Medicina, Universidad de la República, Montevideo, Uruguay; f Department of Physiological Sciences, Institute of Biological Sciences, University of Brasília, Brasília, Brazil

**Keywords:** Diphenyl diselenide, Glutathione peroxidase, Mitochondrial dysfunction, Oxidative stress, Antioxidant, HT22 cells, Tert-BuOOH

## Abstract

Oxidative stress and mitochondrial dysfunction are critical events in neurodegenerative diseases; therefore, molecules that increase cellular antioxidant defenses represent a future pharmacologic strategy to counteract such conditions. The aim of this study was to investigate the potential protective effect of (PhSe)_2_ on mouse hippocampal cell line (HT22) exposed to tert-BuOOH (*in vitro* model of oxidative stress), as well as to elucidate potential mechanisms underlying this protection. Our results showed that tert-BuOOH caused time- and concentration-dependent cytotoxicity, which was preceded by increased oxidants production and mitochondrial dysfunction. (PhSe)_2_ pre-incubation significantly prevented these cytotoxic events and the observed protective effects were paralleled by the upregulation of the cellular glutathione-dependent antioxidant system: (PhSe)_2_ increased GSH levels (> 60%), GPx activity (6.9-fold) and the mRNA expression of antioxidant enzymes *Gpx1* (3.9-fold) and *Gclc* (2.3-fold). Of note, the cytoprotective effect of (PhSe)_2_ was significantly decreased when cells were treated with mercaptosuccinic acid, an inhibitor of GPx, indicating the involvement of GPx modulation in the observed protective effect. In summary, the present findings bring out a new action mechanism concerning the antioxidant properties of (PhSe)_2_. The observed upregulation of the glutathione-dependent antioxidant system represents a future pharmacologic possibility that goes beyond the well-known thiol-peroxidase activity of this compound.

## Highlights

• (PhSe)_2_ protects HT22 cells against cell death induced by tert-BuOOH.
• (PhSe)_2_ prevents the oxidative stress and mitochondrial dysfunction induced by tert-BuOOH.
• (PhSe)_2_ increases GPx activity and GSH levels in HT22 cells.
• The modulation of GPx is involved in the protective effect of (PhSe)_2_.

## Graphical abstract

**Figure:**
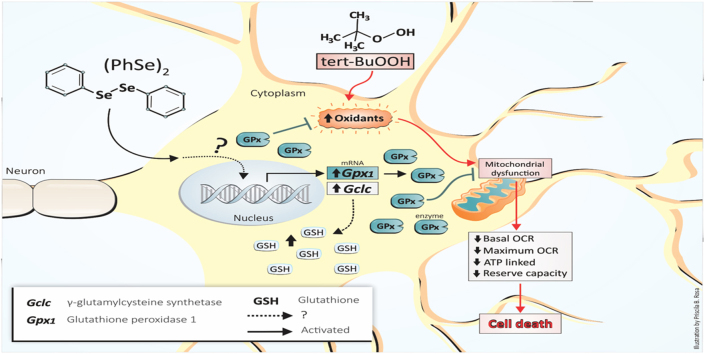

## 1 Introduction

Neurodegenerative diseases, including Alzheimer's disease, Parkinson's disease and amyotrophic lateral sclerosis, differ widely in their pathology and symptoms. However, they share common events that can mediate the neurodegeneration observed in these conditions. Because neurons are highly demanding energy cells that rely on the mitochondrial integrity to support this bioenergetic demand [1], mitochondrial dyshomeostasis represent one of the mentioned common events mediating the neuronal damage characteristic of different neurodegenerative diseases [2].

The brain is particularly vulnerable to oxidative stress because of its high metabolic rate and relatively low antioxidant defense capability [3]. Because the brain is rich in lipids containing polyunsaturated fatty acids (PUFAs), lipid peroxidation is the prominent type of oxidative damage [4]. In this context, glutathione peroxidases (GPxs), which catalyze the reduction of H_2_O_2_ or organic hydroperoxides to water or the corresponding alcohols, respectively, typically using glutathione (GSH) as reductant, play a critical role in the control of oxidative stress in the brain [5]. Particularly, GPx1, which is present in both neurons and glial cells [6], is ubiquitously found in the cytosol and mitochondria of cells, working in the water phase. GPx4 is a plasma and mitochondria membrane-associated enzyme, where it catalyzes the reduction of lipid hydroperoxides [5], [7]. Moreover, overexpression of GPx decreases neuronal cell death and reduces hydrogen peroxide accumulation and the consequent lipid peroxidation under neurotoxic conditions [8].

Taking into account the aforementioned, strategies to counteract harmful cellular oxidative events and preserve mitochondrial integrity by increasing cellular antioxidant defenses are emerging as promising therapeutic approaches to prevent neuronal damage. In this scenarium, various organoselenium compounds have been synthesized and studied over the years aiming to mimic the peroxidase activity of the GPx, however the biological properties of organoselenium compounds are much more complex and seem to go far beyond their GPx mimetic activity [9]. Of particular importance, our group and others are dedicated to evaluating the antioxidant properties of the simple diaryldiselenide, diphenyl diselenide (PhSe)_2_. Pharmacological properties of (PhSe)_2_ has been described in experimental models of central nervous system (CNS) pathologies, including Parkinson's disease [10], Alzheimer's disease [11], methylmercury-induced neurotoxicity [12], and as antidepressive therapy [13]. Some *in vitro* studies were performed to evaluate the mechanisms involved in the cytoprotective effect of (PhSe)_2_ against different oxidative stress conditions. (PhSe)₂ prevented the endothelial and mitochondrial dysfunction induced by peroxynitrite through enhancing cellular antioxidant defenses [14], [15]. Moreover, this simple organoselenium compound protected macrophages, against the oxLDL cytotoxic effects by reducing the oxidants production, which in turn prevented the nuclear factor NF-κB activation [16].

As already mentioned, specific organoselenium compounds have been synthesized to mimic the peroxidase activity of the GPx and therefore protect against oxidative stress-related conditions [17]. However, the simple thiol-peroxidase activity of these compounds seem to be not enough to justify their antioxidant properties in biological systems [17], [18]. In this study, we aimed to evaluate the beneficial effects of (PhSe)_2_ against oxidative changes promoted by tert-BuOOH in the HT22 neuronal cell line. The hippocampal neuronal cell line HT22 has been used to unravel mechanistic aspects associated with hippocampal damage and potential therapeutic strategies in neurodegenerative diseases [19] while tert-Butyl hydroperoxide (tert-BuOOH) has been widely used to induce oxidative stress and mitochondrial dysfunction in a variety of cell types including HT22 cell [20]. Our data indicate that (PhSe)_2_ was effective in preventing tert-BuOOH-induced oxidants production and mitochondrial dysfunction by modulating the glutathione-dependent antioxidant system, particularly the GPx1.

## 2 Material and methods

### 2.1 Reagents

β-Nicotinamide adenine dinucleotide phosphate sodium salt reduced (NADPH), dimethyl sulfoxide (DMSO), glutathione reductase from baker's yeast, reduced glutathione, 3-(4,5-dimethylthiazol- 2-yl)-2,5-diphenyltetrazolium bromide (MTT), propidium iodide (PI), 2′,7′-dichlorofluorescein diacetate (DCFH_2_-DA), 5,5′-dithiobis-(2- nitrobenzoic-acid) (DTNB), *tert*-butyl hydroperoxide (tBuOOH), mercaptosuccinic acid (MS), proton ionophore carbonyl cyanide 4-(trifluoromethoxy) phenylhydrazone (FCCP), antimycin A and oligomycin were purchased from Sigma-Aldrich (St. Louis, MO, USA). Dulbecco's Modified Eagle's Medium (DMEM) and fetal bovine serum (FBS) were obtained from Gibco Life Technologies (Carlsbad, CA). MitoSOX™ Red probe was obtained from Invitrogen (USA). RQ1 RNase-Free DNase, GoScript™ Reverse Transcription System and GoTaq qPCR Master Mix were purchased from Promega Corporation (USA). All other chemicals were of the highest grade available commercially.

### 2.2 Cell culture

The HT22 cells, an immortalized mouse hippocampal neuronal cell line, were a gift from Dr. David Schubert (Salk Institute, La Jolla, CA, USA). HT22 cells were maintained in DMEM supplemented with 5% (v/v) fetal bovine serum (FBS; Gibco/lnvitrogen), 2 mM glutamine, 100 units/mL penicillin, 100 μg/mL streptomycin, 10 mM Hepes, 24 mM glucose, 44 mM NaHCO_3_ and incubated at 37 °C in a humidified atmosphere of 5% CO_2_. Cells were subcultured at confluences of 80–90%, and used between the 3rd and 12th passages. Cell suspensions were seeded in dish plates (100 ×20 mm), 96-, or 6-well plaques at different cell densities, depending on the experimental procedure, as described below.

### 2.3 Cell viability assays

Cell viability was measured by two different assays: MTT reduction and PI uptake. For these assays, cells were plated into 96-well plates at a cell density of 1 × 10^3^ cells/well and cultivated for 24 h. In order to determine the tBuOOH toxicity, HT22 cells were incubated with 0.05% DMSO (used as vehicle in further experiments) and maintained in culture for additional 48 h. Then, cells were exposed to tBuOOH (10, 20, 40, 70 and 100 μM) for 12 h; - to determine the timeline of tBuOOH toxicity, cells were incubated with tBuOOH (40 μM) for 2, 4, 5, 6, 7, 9 and 12 h. In parallel experiments, cells were incubated with (PhSe)_2_ (0.5, 1, 3, 6 and 10 μM) for 48 h in order to evaluate the concentration-response effect of (PhSe)_2_ *per se*; – to evaluate the protective effect of (PhSe)_2_, cells were pre-incubated with nontoxic concentration of (PhSe)_2_ (0.5, 1, 2 and 5 μM) or vehicle (DMSO, 0.05%) during 48 h. Afterward, the medium was replaced by a fresh medium and cells were exposed to tBuOOH (40 μM) for 12 h. To establish potential mechanisms involved in the cytoprotective effect of (PhSe)_2_, cells were pre-treated with (PhSe)_2_ (2 μM) or vehicle (DMSO, 0.05%) during 48 h and then 5 mM mercaptosuccinic acid (MS; inhibitor the GPx activity) was added 30 min before the exposure to tBuOOH (40, 70 and 100 μM) for additional 12 h. tBuOOH was diluted in water and always prepared at the time of use. The reduction of 3-(4, 5-dimethylthiazol-2-yl)-2, 5- diphenyl-tetrazolium bromide (MTT) to the formazan product by mitochondrial dehydrogenases in viable cells was conducted as described by Mosmann [27]. Propidium iodide (PI), which is excluded by living cells but rapidly enters cells with damaged membranes and binds to DNA, rendering them brightly fluorescent, was measured according to Riccardi and Nicoletti [28]. Results of MTT assays were expressed as percentage of untreated cells and the results of PI uptake assays were expressed as percentage of 2% Triton X-100-treated cells that represent the 100% of death. All experiments were performed in triplicate and read on a spectraMax Paradigm spectrofluorometer (Molecular Devices).

### 2.4 Measurement of oxidants production

Intracellular oxidants production was detected using the 2′,7′- dichlorodihydrofluorescein diacetate (DCFH_2_-DA) and mitochondrial superoxide radical generation was measured using MitoSOX probe. HT22 cells (1 × 10^3^ cells/well) were plated into 96-well plates for 24 h and then pre-incubated with (PhSe)_2_ (2 μM) or vehicle (DMSO, 0.05%) for 48 h. Afterward, DCFH_2_-DA (1 μM) was added to the culture medium and incubated for 30 min, at 37 °C in a humidified atmosphere of 5% CO_2_. In parallel, MitoSOX (5 μM) was dissolved in medium HBSS and was added in HT22 cells pre-incubated with (PhSe)_2_, and incubated for 15 min at 37 °C. In both experiments, the medium was removed and fresh Hanks’ balanced salt solution (HBSS) was added, and then cells were treated with tBuOOH (40, 70 and 100 μM). The fluorescence of DCF (485 nm excitation and 520 nm emission) inside the cells was immediately measured after tBuOOH addition (during 2 h at each 10 min) and used to evaluate the oxidants production. Results were expressed as Area Under the Curve (A.U.C) and compared to the control (vehicle). The fluorescence of MitoSOX (510 nm excitation and 580 nm emission) was immediately measured after tBuOOH (40, 70 and 100 μM) addition (in 4 h) and the results were expressed as percentage of untreated cells. All experiment were performed in triplicate and read on a spectraMax Paradigm spectrofluorometer (Molecular Devices).

### 2.5 High-resolution respirometry of intact cells

To evaluate the mitochondrial oxygen consumption, HT22 cells (3 × 10^5^ cells/plate) were plated into Petri plates for 24 h. After, cells were pre-incubated with (PhSe)_2_ (2 μM) or vehicle for 48 h followed by tBuOOH (40 μM) exposure for 2 h or 4 h. Approximately one million suspended cells (in DMEM 5% FBS) were charged in OROBOROS Oxygraph-2k chambers under continuous stirring at 750 r.p.m. at 37 °C. After stabilization of the signal, basal oxygen consumption rates (OCR) was recorded, which is defined as respiration with the physiological substrates in growth medium. ATP synthase was inhibited with oligomycin (1.25 μM) and uncoupled OCR was recorded. Then, the proton ionophore carbonyl cyanide 4-(trifluoromethoxy) phenylhydrazone (FCCP, uncoupler of oxidative phosphorylation) was used in 0.5 μM steps to determination the maximum OCR or maximum respiratory capacity. Respiration was inhibited by application of 2.5 μM antimycin A to determine the non-mitochondrial OCR (residual oxygen consumption – ROX). DatLab software (Oroboros Instruments) was used for data acquisition and analysis. The difference in OCR was compared with the control group (vehicle).

### 2.6 Assessment of glutathione peroxidase (GPx) activity

HT22 cells (1 × 10^5^ cells/well) were seeded for 24 h in 6-well plates and pre-incubated with (PhSe)_2_ (2 μM) or vehicle (DMSO, 0.05%) for additional 48 h. In another experimental set, cells were pre-incubated with (PhSe)_2_ (2 μM) or vehicle (DMSO, 0.05%) for 43 h and then exposed to Mercaptosuccinic acid (MS; 5 mM) for additional 12 h. After treatments, the medium was aspirated and the cells were washed once with phosphate-buffered saline (PBS), trypsinized and suspended (1:1 ratio) in DMEM 5% FBS. Cell suspension were centrifuged at 500 ×*g* for 2 min at room temperature and the cell pellets were stored at − 80 °C until assay. For GPx assay, cell pellets were suspended in 50 μL of buffer (20 mM TrisHCl, 0.25 M sucrose; containing 0.4 mM β-mercaptoethanol) at pH 7.4 on ice. The samples were sonicated for 5 min (three times) on ice with vortex of 20 s to each sonicate time, and centrifuged at 10,000×*g* for 15 min at 4 °C. The supernatant was collected and used for kinetic GPx activity assay (10 μL/well). GPx activity was performed by measuring the consumption of NADPH at 340 nm [21] and optimized conditions for HT22 cell lysate described by Panee et al. [22]. The following reagents and concentrations were used: tert-butyl hydroperoxide (0.32 mM), GSH (1.88 mM), GR (84 mU/mL), EDTA (1 mM), NaN_3_ (1 mM), NADPH (0.2 mM) and Tris-HCl pH 7.6 (0.1 M). The experiments were performed in triplicate and read on a spectraMax Paradigm Multi-Mode Microplate Reader (Molecular Devices). The results were expressed as nmol NADPH consumed per min per milligram of protein.

### 2.7 Determination of glutathione (GSH) and nonproteic thiols (NPSH) content

GSH and NPSH content were determined using a fluorimetric assay described by Hissin and Hilf [23] and a spectrophotometric assay as described by Ellman [24], respectively. HT22 cells (1 × 10^5^ cells/well) were seeded for 24 h in 6-well plates and incubated with (PhSe)_2_ (2 μM) or vehicle (DMSO, 0.05%) for 48 h. Then, cells were harvested in 150 μL of PBS buffer (0.05% Triton X-100, pH 7.4) and mixed in a trichloroacetic acid 10% solution. After centrifugation (5000×*g* at 4 °C for 10 min), supernatant was used to determined GSH and NPSH content. A volume of 30 μL of supernatant was incubated with 10 μL of ortho-phthalaldehyde (0.1% w/v in methanol) and 160 μL of 100 mM Na_2_HPO_4_ for 15 min at room temperature to fluorimetric assay. A volume of 50 μL of supernatant was incubated with 25 μL of DTNB (10 mM) and 125 μL of potassium phosphate buffer (1 M) for 15 min at room temperature to spectrophotometry assay. Fluorescence intensity (350 nm excitation and 420 nm emission) and spectrophotometry (absorbance 412 nm) assay were read on a spectraMax Paradigm Multi-Mode Microplate Reader (Molecular Devices). Cellular GSH and NPSH contents were calculated by using concurrently run standard curve of GSH. The results were expressed as nmol GSH per milligram of protein or percent of control group (vehicle).

### 2.8 Protein quantification

Protein quantification was performed by Lowry method [25], using bovine serum albumin as standard.

### 2.9 Transcript level of antioxidant enzymes by RT-qPCR

To evaluate the transcript level of *Gpx1, γ-glutamylcysteine synthethase (Gclc)*, *Gpx4*, heme oxygenase-1 (*HO-1*), catalase (*Cat*), *superoxide dismutase-2 (Sod2), thioredoxin reductase 2 (Txnrd2), and peroxiredoxins isoforms (Prdx2, Prdx3 and Prdx5)*, HT22 cells (1 × 10^5^ cells/well) were seeded in 6-well plates and cultivated for 24 h. Then, cells were incubated with (PhSe)_2_ (2 μM) or vehicle (DMSO, 0.05%) during 30 h. Total RNA was extracted in different times of incubation (3 h, 6 h, 12 h, 24 h and 30 h) using Tri-reagent (Sigma-Aldrich) according to the manufacturer's instructions. DNA contamination of samples was removed by treatment with RQ1 RNase-Free DNase (Promega) at 25 °C for 1 h. After, RNA samples (90 μL) were precipitated with 10 μL acetate sodium (3 M) and 100 μL of isopropyl alcohol at room temperature for 10 min. After the centrifugation at 12,000×*g* for 10 min, the pellet was washed with 70% ethanol and centrifuged at 12,000×*g* for 10 min. PCR of *β-act* gene was realized for verify the effectiveness of DNase treatment. RNA concentrations and quality were determined using spectrophotometry (BIO-5000-BI/ KASUAKI). RNA samples with A260/280 and A260/230 absorbance ratio > 1.8 were considered acceptable for cDNA synthesis. The integrity of total RNA was determined by 1.5% agarose gel electrophoresis. For cDNA synthesis, GoScript™ Reverse Transcription System (Promega) were used according to the manufacturer's instructions. The reaction (20 μL) consisted of 1 µg of total RNA, Oligo(dT)_15_ primer (0.25 µg), 4 μL of GoScript 5X Reaction Buffer, GoScript Reverse Transcriptase (160 U, Promega), 0.5 mM of each dNTP, 2.5 mM MgCl_2_, and ribonuclease inhibitor (20 U). First, RNA samples and Oligo(dT)_15_ were mixed, incubated at 70 °C for 5 min and immediately placed on ice for 5 min. After, other mentioned above components were added and runs at 25 °C for 5 min, 42 °C for 60 min, and 70 °C for 15 min.

Real time PCR was carried out in 96-well plates using the 7900HT Fast Real-Time PCR System (Applied Biosystems), and was performed with GoTaq qPCR Master Mix (Promega). Sequences of primers of the genes are indicated in the Table 1. The qPCR reaction (10 μL) consisted of 1 μL of 10-fold diluted cDNA, 0.3 μL of each primer (10 µM), 5 μL of master mix (2X), 0.1 μL of CXR and 3.3 μL DEPC-water. The thermal cycling program was as follows: 50 °C for 2 min, 95 °C for 10 min, 40 amplification cycles of 95 °C for 15 s and 60 °C or 62 °C for 1 min, and a melting curve analysis at 95 °C for 15 s, 60 °C for 15 s, and 95 °C for 15 s. Each sample was analyzed in technical duplicate. The amplification efficiency (E) was calculated according to the equation E = 10^(1/−slope)^ from a standard curve of five-fold serial dilutions (1/10, 1/20, 1/40, 1/80, 1/160) of pooled cDNA. Efficiency of 1.9–2.1 and Pearson's coefficients of determination (R^2^) of each gene > 0.99 were considered for RT-qPCR. *β-act* gene was used to normalize the transcript levels of genes, and calculated by the 2^-ΔΔCT^ method [26]. To determine the copies number of *Gpx4*, a ten-fold series dilution (10^8^ to 10^4^ copies) of pGEM-T plasmid carrying the fragment of *mtGpx4* (mitochondrial *Gpx4*) or *cGpx4* (cytoplasmic *Gpx4*) or *Gapdh* was made and used as standard curves for absolute quantification by qPCR [27].

**Table 1:** Primer sequences used for RT-qPCR experiments.

**Gene**		**Primer sequence (5′–3′)**	**Amplicon size (bp)**	**AT**	**Reference**
**Symbol**	**Name**			**(°C)**	
***Gpx1***	*Glutathione peroxidase 1*	CTCACCCGCTCTTTACCTTC	129	60	Quispe et al. [20]
		CAAAGTTCCAGGCAATGTCG			
***mtGpx4***	*Mitochondrial glutathione peroxidase 4*	CCGCCGAGATGAGCTGG	158	60	Casañas-Sánchez et al. [28]; Quispe et al. [20]
		GTCGATGTCCTTGGCTGAG			
***mcGpx4***	*Mitochondrial and cytosolic glutathione peroxidase 4*	GTCTGGCAGGCACCATGT	83	60	Quispe et al. [20]
		GTCGATGTCCTTGGCTGAG			
***Gclc***	*Glutamate-cysteine ligase catalytic subunit*	TTACCGAGGCTACGTGTCAGAC	200	62	Kurauchi et al. [29]
		TGTCGATGGTCAGGTCGATGTC			
***HO-1***	*Heme oxygenase 1*	CAGCCCCACCAAGTTCAAA	63	60	Tighe et al. [30]
		TCAGGTGTCATCTCCAGAGTGTTC			
***Cat***	*Catalase*	TGAAGCAGTGGAAGGAGCAG	156	62	This study
		AGTGTGCCATCTCGTCAGTG			
***Sod2***	*Superoxide dismutase 2*	GACCTGCCTTACGACTATG	108	60	This study
		GGTGGCGTTGAGATTGTTC			
***Prdx2***	*Peroxiredoxin 2*	CTTTTGTTTGCCCCACGGAG	244	62	This study
		AGACCCCTGTAAGCAATGCC			
***Prdx3***	*Peroxiredoxin 3*	AAGGCGTTCCAGTTTGTAG	88	60	This study
		CTGTTGGACTTGGCTTGA			
***Prdx5***	*Peroxiredoxin 5*	GTGGCCTGTCTGAGCGTTAA	170	60	This study
		TTCAGCCGACGATTCCCAAA			
***Txnrd2***	*Thioredoxin reductase 2*	GATCAAGTGTGGGGCTTCA	164	60	Quispe et al. [20]
		GTGTCCTTAGCTCAGCAGGG			
***β-act***	*β-actin*	CATTGCTGACAGGATGCAGAAGG TGCTGGAAGGTGGACAGTGAGG	138	60	Flowers et al. [31]
***Gapdh***	*Gliceraldehyde-3-phosphate dehydrogenase*	CATCACTGCCACCCAGAAGACTG	153	60	Know et al. [32]
		ATGCCAGTGAGCTTCCCGTTCAG			

AT: Annealing temperature.

### 2.10 Statistical analysis

Statistical analysis of the data was performed using the STATISTICA software system, version 8.0. (StatSoft, Inc., 2008). Normal (Gaussian) distribution and homogeneity of variance were evaluated with the Kolmogorov–Smirnov test and Levene's test, respectively. Significant differences were evaluated by Student's *t*-test, one-way, two-way or three-way analysis of variance (ANOVA), depending on the experimental design. Multiple comparisons were performed using the Tukey *post-hoc* test. Results were expressed as mean ± SEM. *p* < 0.05 was considered statistically significant. All graphics were made using the GraphPad PRISM® software version 6.00 for Windows (GraphPad Software, San Diego, CA, USA).

## 3 Results

### 3.1 tert-BuOOH induces toxicity in HT22 cells

**Fig. 1:**
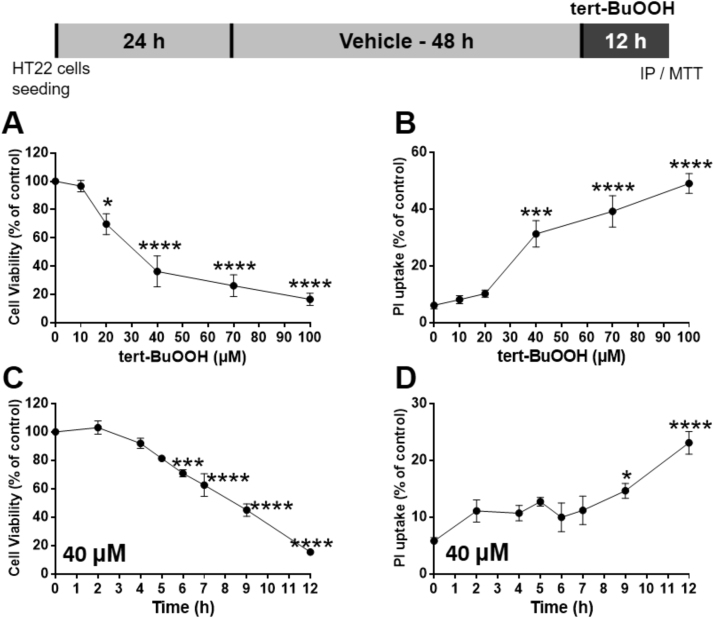
*tert*-BuOOH induced toxicity in HT22 cells. HT22 cells were exposed to different tert-BuOOH concentrations (10–100 μM) for ~ 12 h (A, B) or to 40 μM for 2–12 h (C, D). Cell viability was evaluated by MTT reduction (A, C) and PI uptake (B, D). Results of MTT assay were expressed as the percentage of MTT reduction with respect to control group. Results of PI assays were expressed as the percent of PI uptake-cell, where the 100% of cell death value represents cells treated with 2% Triton X-100 during 15 min. Data were represented as mean ± SEM (A,B; n = 6) (C, D; n = 4). * *p* < 0.05, *** *p* < 0.001 and **** *p* < 0.0001 indicate statistical difference from control group by one-way ANOVA, followed by the Tukey *post hoc* test.

The effect of tert-BuOOH exposure in HT22 cell viability was evaluated in time- and concentration-response experiments. The Fig. 1A and B depict concentration-response studies after overnight exposure (~ 12 h) of HT22 cells to tert-BuOOH (0–100 μM). The results show that tert-BuOOH caused a significant decline in the MTT reduction starting at 20 μM (Fig. 1A). In addition, tert-BuOOH induced a disruption in cell plasma membrane (PI uptake) starting at 40 μM (Fig. 1B). Therefore, the 40 μM tert-BuOOH concentration was used in the time-response study (2–12 h). At this condition, the cells present a significant reduction in the ability to convert MTT to formazan starting 6 h after exposures (Fig. 1C) and a significant disruption of the cellular plasma membrane was verified after 9 h (increased PI uptake) (Fig. 1D). Based on these results, it can be argued that cell viability and cell plasma membrane integrity were not significantly affected within two or four hours of exposure to tert-BuOOH (40 μM). Thus, these conditions were chosen to investigate molecular mechanisms mediating the toxicity induced by tert-BuOOH, as well as the potential protective effects of (PhSe)_2_.

### 3.2 (PhSe)_2_ protects HT22 cells against tert-BuOOH-mediated toxicity

**Fig. 2:**
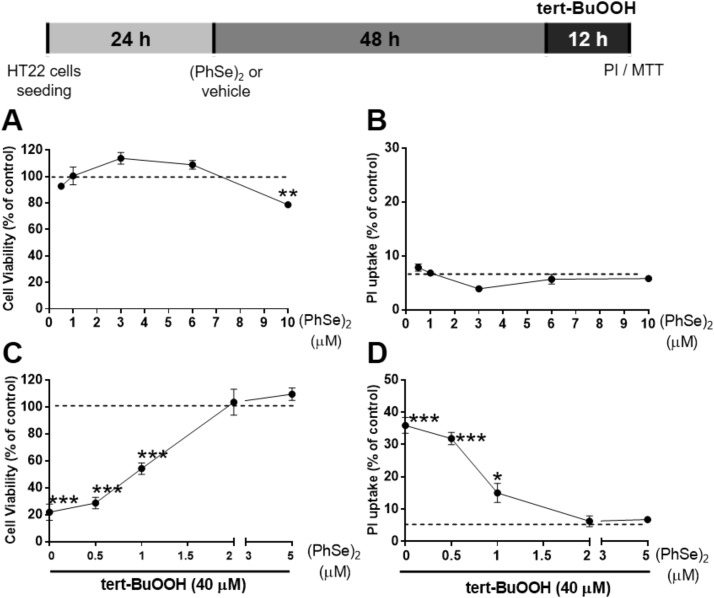
(PhSe)_2_ protects cells against tert-BuOOH-mediated toxicity. (A-B) HT22 cells were pre-incubated with different concentrations of (PhSe)_2_ (0.5, 1, 3, 6 and 10 μM) for 48 h. Cell viability and PI uptake were evaluated by MTT reduction and PI uptake, respectively. (C-D) Cells were pre-incubated with non-toxic concentrations of (PhSe)_2_ (0.5; 1; 2 and 5 μM) for 48 h followed by exposure to tert-BuOOH (40 μM) for additional 12 h. Cell viability was evaluated by MTT reduction (A; C) and cell death by PI uptake (B; D). Results of MTT assay were expressed as the percentage of MTT reduction with respect to control group. Results of PI assays are expressed as percent of PI uptake, where the 100% of cell death value represent cells treated with 2% Triton X-100 during 15 min. Data are represented as mean ± SEM (A-B; n = 4, C-D; n = 6). * *p* < 0.05, ** *p* < 0.01, *** *p* < 0.001 indicate statistical difference from control group (dashed line) by one-way ANOVA followed by the Tukey *post hoc* test.

Firstly, to assess non-toxic concentrations of (PhSe)_2_, a concentration-response study was conducted by pre-incubating HT22 cells for 48 h with concentrations of (PhSe)_2_ ranging from 0.5 to 10 μM. As shown in Fig. 2A-B, there was no significant decrease in cell viability after 48 h of pre-incubation with (PhSe)_2_ at concentrations up to 6 μM. Based on these results, the non-toxic concentrations of (PhSe)_2_ (1–5 μM) were chosen to evaluate its protective effect against tert-BuOOH-mediated cytotoxicity (Fig. 2A-B). Our results show that the pre-incubation with 2 μM of (PhSe)_2_ for 48 h significantly protected HT22 cells against tert-BuOOH-induced decrease in MTT reduction (Fig. 2C) and increase PI uptake (Fig. 2D).

### 3.3 (PhSe)_2_ prevents tert-BuOOH-induced oxidants production in HT22 cells

**Fig. 3:**
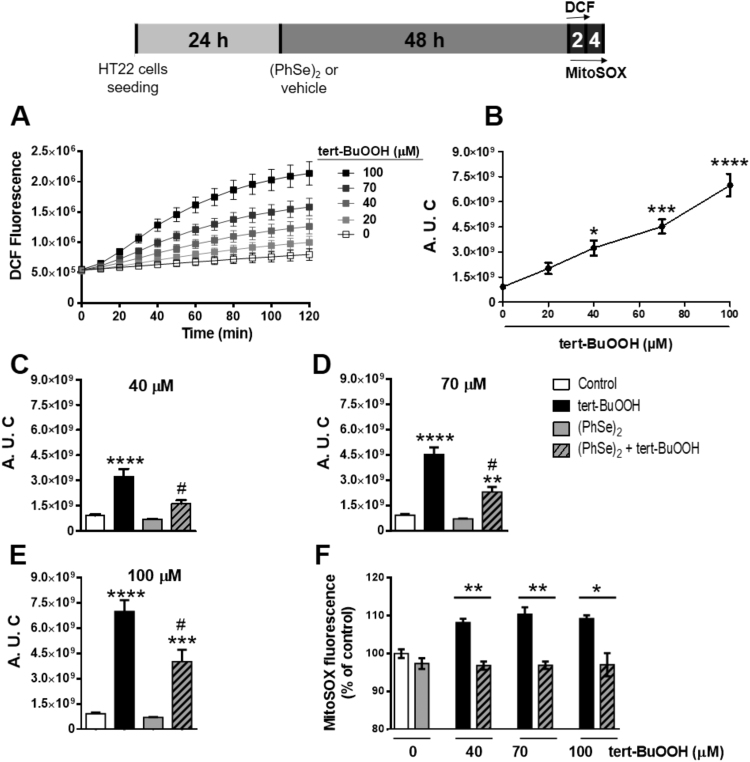
(PhSe)_2_ prevents oxidants production induced by tert-BuOOH. (A) HT22 cells were exposed to tert-BuOOH followed by kinetic oxidation of DCFH_2_-DA (1 μM) monitored for 2 h. Representative time course of oxidants production in HT22 cells exposed to different concentrations of tert-BuOOH (20, 40, 70 and 100 μM). (B) The kinetic oxidation of DCFH_2_-DA (1 μM) was monitored for 2 h. Area under the curve (A. U. C) was calculated from the kinetic data shown in figure A. (C-F) HT22 cells were pre-incubated with 2 μM of (PhSe)_2_ or vehicle (control) for 48 h and then exposed to (C) 40 μM, (D) 70 μM, and (E) 100 μM tert-BuOOH. (F) MitoSOX assay was recorded after 4 h of tert-BuOOH exposure. Data are represented as mean±SEM (n = 5) * *p* < 0.05; ** *p* < 0.01; *** *p* < 0.001; **** *p* < 0.0001 indicate the statistical differences compared to the control group (white bar), ^#^*p* < 0.001 indicate the differences between (PhSe)_2_ and control group that were exposed to tert-BuOOH (black bar). One-way (B), Student's *t*-test (F) or two-way (C-E) ANOVA followed by the Tukey *post hoc* test were realized.

Taking into account that tert-BuOOH-mediated cell damage is commonly related to oxidative stress, we investigated whether (PhSe)_2_ protects HT22 cells from tert-BuOOH-induced oxidants generation. Oxidants-mediated DCFH_2_ oxidation was evaluated in HT22 cells within the first 2 h after tert-BuOOH exposure (40, 70 and 100 μM). A concentration-dependent increase in oxidants production was observed after tert-BuOOH exposure (Fig. 3A-B). The pre-incubation with (PhSe)_2_ completely prevented the oxidants production in HT22 cells exposed to tert-BuOOH (40 μΜ) (Fig. 3C) and partially prevented at higher tert-BuOOH concentrations (70–100 μM) (Fig. 3D-E). Of note, oxidants production was not changed in HT22 cells pre-incubated only with (PhSe)_2_ (Fig. 3C-E). In accordance, our results using MitoSOX showed that the pre-incubation with (PhSe)_2_ (2 μM) for 48 h prevented the peroxide-induced mitochondrial oxidants generation after 4 h of exposure to tert-BuOOH concentrations (40–100 μM) (Fig. 3F).

### 3.4 (PhSe)_2_ prevents the mitochondrial dysfunction induced by tert-BuOOH

**Fig. 4:**
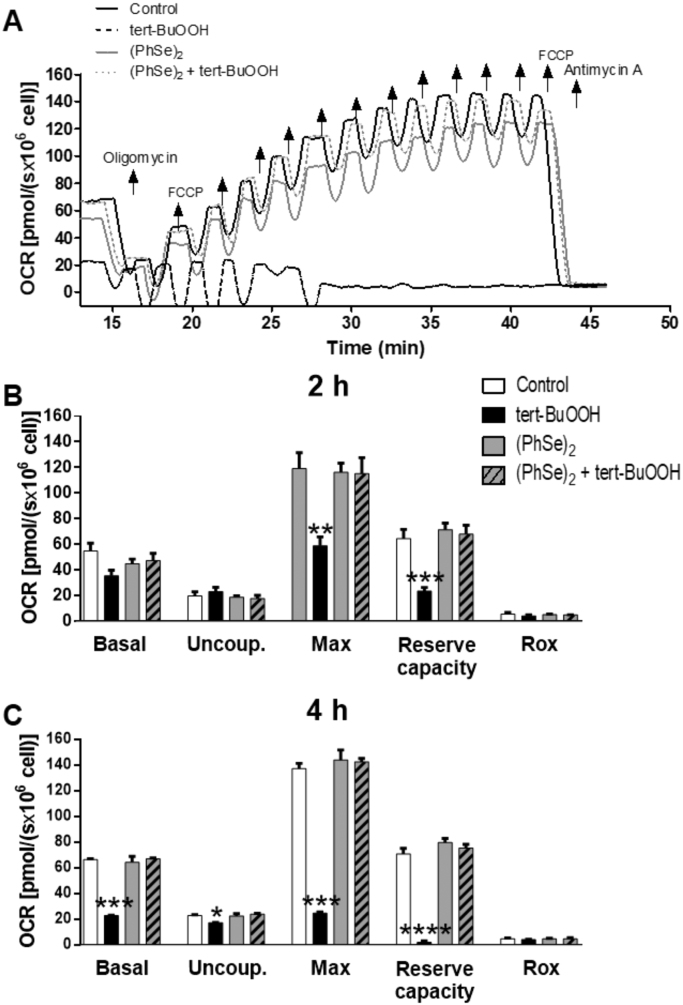
(PhSe)_2_ prevents the mitochondrial dysfunction induced by tert-BuOOH (A) Representative respirometry assay of intact HT22 cells pre-incubated with 2 μM (PhSe)_2_ for 48 h followed the exposure or not with tert-BuOOH (40 μM) for additional 4 h. After OCR stabilization, the following electron transport system (ETS) modulators were added: Oligomycin (1.25 μM) to measure uncoupled respiration; sequential additions (0.5 μM) of FCCP to achieve maximum respiration; and antimycin A (2.5 μM) to determine residual oxygen consumption rates. OCR records and mitochondrial reserve capacity of HT22 cells exposed or not with tert-BuOOH (40 μM) for 2 h (B) and 4 h (C) were evaluated in DMEM-5% FBS in the following conditions: basal respiration; after inhibition of ATP synthase with oligomycin (uncoupled respiration – Uncoup.); upon titration with FCCP (maximum respiration – Max); and after inhibition of the respiratory complex III with antimycin A (residual respiration – Rox). Data are represented as mean ± SEM (n = 4) * *p* < 0.05; ** *p* < 0.01; *** *p* < 0.001 and **** *p* < 0.0001 indicate statistical differences compared to the control group (white bar). Two-way ANOVA followed by the Tukey *post hoc* test was realized.

Using high-resolution respirometry, the mitochondrial oxygen consumption in intact HT22 cells was evaluated. The exposure of HT22 cells for 2 or 4 h to 40 μM tert-BuOOH, concentration which not affect the cell viability (Fig. 1C-D), caused a significant decrease in several mitochondrial oxygen consumption rates (OCR) (Fig. 4A). Already in a short period of tert-BuOOH exposure (2 h), a significant decrease in the maximal respiration rate and mitochondrial reserve capacity were observed in HT22 cells pre-incubated with vehicle (DMSO, 0.05%) (black bar) (Fig. 4B). Mitochondrial dysfunction was more evident after 4 h of tert-BuOOH exposure (compared with 2 h exposure), characterized by a decrease in the basal OCR, the ATP-linked oxygen consumption, the maximum OCR and mitochondrial reserve capacity (black bar) (Fig. 4C). The pre-incubation with 2 μM (PhSe)_2_ prevented the mitochondrial dysfunction induced by tert-BuOOH in HT22 cells (Fig. 4).

### 3.5 (PhSe)_2_ modulates the glutathione-dependent antioxidant system in HT22 cells

**Fig. 5:**
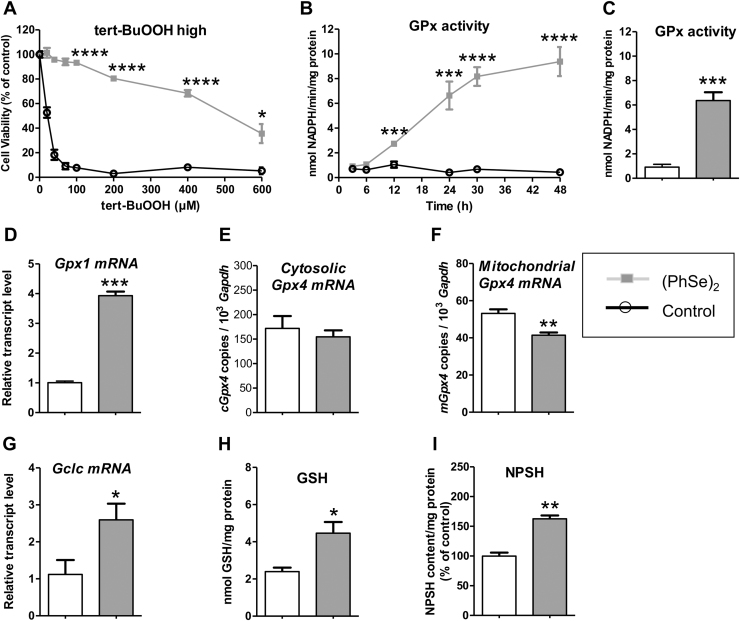
(PhSe)_2_ modulates the glutathione-dependent antioxidant system in HT22 cells. HT22 cells were pre-incubated with 2 μM (PhSe)_2_ for 48 h followed exposure to tert-BuOOH concentrations (20–100 µM) (A). HT22 cells were treated with 2 μM (PhSe)_2_ for 48 h to determination of GPx activity (B-C), GSH content (H), and NPSH content (I). Moreover, *Gpx* isoforms (D, E and F) and *Gclc* (G) gene expression was evaluated after 30 h of incubation with 2 μM (PhSe)_2_. Cellular GPx activity is expressed as nmol NADPH consumed per minute per mg of protein. Transcripts levels of genes were normalized with *β-actin* gene and calculated by the 2^−ΔΔCT^ method. Copies number of *Gpx4* transcripts of mitochondrial and cytosolic *Gpx4* isoforms were calculated in relation of number copies of *Gapdh* using standard curve of plasmids containing these genes. Values are represented as the mean ± SEM (A; n = 4, B-C; n = 6, D-G, n = 4; H, n = 4, I; n = 3). * *p* < 0.05; ** *p* < 0.01; *** *p* < 0.001 and **** *p* < 0.0001 indicate statistical difference from control group (Student's *t*-test).

Results from Fig. 5A strongly suggested that (PhSe)_2_-treated cells were more efficient to neutralize tert-BuOOH. The results show an intense tert-BuOOH-induced decrease in cell viability since concentration as 20 μM, while (PhSe)_2_-pre-incubated cells resist to the cell death induced by tert-BuOOH even in high peroxide concentrations tested (20, 40, 70, 100, 200, 400 and 600 μM). Because the glutathione-dependent antioxidant system plays a key role in tert-BuOOH detoxification, we investigated whether this system, particularly GPx activity, GSH levels, *Gpx* isoforms and *Gclc* gene expression, could be modulated by (PhSe)_2_. We found a time-response effect of (PhSe)_2_ on its ability to increase the GPx activity (Fig. 5B), reaching a significant increase after 48 h of (PhSe)_2_ incubation (around 6–8 fold increase – Fig. 5C). In order to investigate if the (PhSe)_2_-induced increase in the GPx activity and GSH levels was correlated with the enzyme gene expression of *Gpx1*, *Gpx4*, and *Gclc*, the quantitative PCR analyses of gene expression at 30 h were performed. Our results show a significant increase in the *Gpx1* expression after 30 h of (PhSe)_2_ incubation (around 4 fold increase – Fig. 5D). The copies number of *cGpx4* and *mtGpx4* transcripts were not upregulated by (PhSe)_2_ (Fig. 5E-F). Regarding GSH modulation, the results show that (PhSe)_2_ caused a significant increase in the GSH (86.2%) and NPSH (62.5%) levels (Fig. 5H-I) that was accompanied by a marked increase (2.3-fold) in the expression of *Gclc*, whose encoded glutamate-cysteine ligase, the rate-limiting enzyme in glutathione biosynthesis (Fig. 5G).

### 3.6 Protective effect of (PhSe)_2_ depends on the GPx activity modulation

**Fig. 6:**
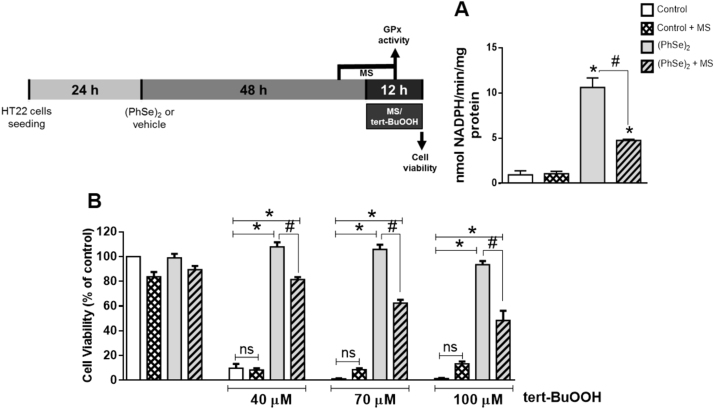
The inhibition of GPx activity by MS reverses the cytoprotective effect of (PhSe)_2_. (A) GPx activity was evaluated in HT22 cells pre-incubated with (PhSe)_2_ (2 μM) for 43 h followed by incubation with mercaptosuccinic acid (MS) (5 mM, GPx inhibitor) for ~12 h. Results are shown as mean ± SEM (n = 3). * *p* < 0.0001 indicate statistical difference from control group (white bar) and ^#^*p* < 0.0001 indicate statistical difference from (PhSe)_2_ group without MS (two-way ANOVA followed by the Tukey *post hoc* test). (B) HT22 cells were pre-incubated with (PhSe)_2_ for 48 h and exposed to tert-BuOOH (40, 70 and 100 μM) for 12 h, in the presence or absence of MS (5 mM). Cell viability was evaluated by MTT reduction. Results are shown as mean±SEM (n = 5). * *p* < 0.0001 indicate statistical difference from control group (white bar) and ^#^*p* < 0.0001 indicate statistical difference between (PhSe)_2_ in the presence or not of MS (three-way ANOVA followed by the Tukey *post hoc* test). The data were compared with their respective controls (white bar) for each tert-BuOOH concentration.

To understand the role of GPx on the protective effect of (PhSe)_2_ against tert-BuOOH-induced toxicity in HT22 cells, we performed an experimental approach using mercaptosuccinic acid (MS), a specific and potent inhibitor of GPx enzyme [36]. HT22 cells were pre-incubated with (PhSe)_2_ (2 μM) in the presence or absence of MS (5 mM) followed by exposure to tert-BuOOH (40, 70 and 100 μM). We first showed that MS caused a significant inhibition (about 50%) in the GPx activity in (PhSe)_2_-pre-incubated cells (Fig. 6A). In addition, the treatment of the cells with MS (5 mM) significantly decreased the protective effect of (PhSe)_2_ against tert-BuOOH-induced toxicity in HT22 cells (Fig. 6B). This event was more evident when HT22 cells were exposed to high concentration of tert-BuOOH used in this study (100 μM) (Fig. 6B). The data show that GPx activity upregulation is involved in the protection of (PhSe)_2_ against tert-BuOOH-induced toxicity in HT22 cells.

### 3.7 Time course expression profiles of antioxidant genes modulated by (PhSe)_2_

**Fig. 7:**
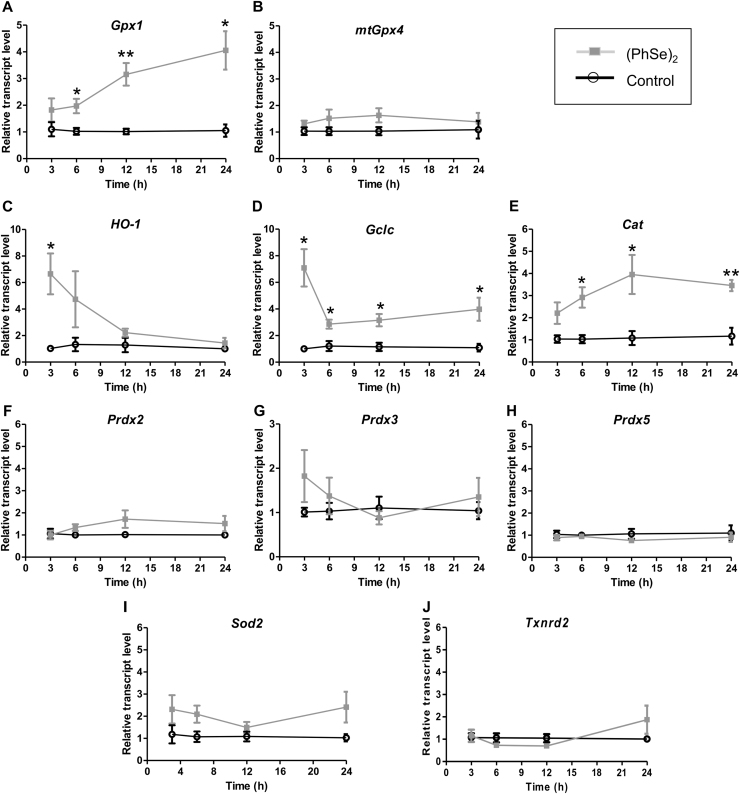
Time course expression profile of antioxidant genes modulated by (PhSe)_2_. HT22 cells were pre-incubated with 2 μM (PhSe)_2_ at different times (3 h, 6 h, 12 h and 24 h) and gene expression was quantified. Transcripts levels of *Gpx1* (A), *mtGpx4* (B), *HO-1* (C), *Gclc* (D), *Cat* (E), *Prdx* isoforms (F, G and H), *Sod2* (I) and *Txndr2* (J) were normalized with *β-actin* gene and calculated by the 2^−ΔΔCT^ method. Values are represented as mean ± SEM (A-J; n = 4). * *p* < 0.05 and ** *p* < 0.01 indicate statistical difference from control group (Student's *t*-test).

To analyze the involvement of Nrf-2 and FoxO-3 transcriptional factors in (PhSe)_2_ cytoprotective actions, we performed a quantitative PCR analyses of temporal expression (up to 24 h) of some target genes of these transcriptional factors. Our results show an early (3 h) and intense increase in heme oxygenase-1 (*HO-1*) expression, a sensitive marker of Nrf-2 activation (Fig. 7C). Other selective genes trigged through Nrf-2 were also improved by the pre-incubation with (PhSe)_2_. Fig. 7(D and E) show a time-dependent increase in catalase (*Cat*) and *Gclc* gene expression after (PhSe)_2_ incubation. The FoxO-3-regulated genes (Fig. F-J), superoxide dismutase-2 (*Sod2*), thioredoxin reductase 2 (*Txnrd2*), and peroxiredoxins isoforms *(Prdx2, Prdx3 and Prdx5*) were not modulated by (PhSe)_2_ in HT22 cells. According to the data of Fig. 7A, (PhSe)_2_ also caused a substantial increase throughout the glutathione antioxidant system and here we observed a sustained and time-dependent increase in *Gpx1* gene expression by (PhSe)_2_. However, the relative expression of *mtGpx4* was not modulated up to 24 h (Fig. 7B).

## 4 Discussion

Neuronal cells are particularly vulnerable to oxidative stress because of its high metabolic rate and relatively low antioxidant defense capability [3]. In these cells, lipid peroxidation, which is stimulated by increased levels of peroxides, can significantly disrupt cellular function and therefore lead to death [4]. Particularly in the brain, enzymes of the GPx family, in association with other peroxidases, orchestrate an adaptive response to oxidative stress by directly reducing peroxides, favoring neuronal survival [5], [33]. In this scenario, a significant effort has been done to develop molecules that mimic the peroxidase activity of the GPx, such as synthetic organoselenium compounds [38]. Among these compounds, (PhSe)_2_, a diselenide GPx mimetic, has shown significant beneficial effects in several models of oxidative stress-related pathologies (for a review, see Nogueira and Rocha [34]. Here, we propose (PhSe)_2_ as a promising therapeutic approach to prevent the neuronal damage related to oxidative stress. Our results showed that the potent cytoprotective action of (PhSe)_2_ in preventing the mitochondrial dysfunction and oxidative stress promoted by tert-BuOOH was due to an induction of an adaptive cellular antioxidant response.

There are several studies reporting that (PhSe)_2_ increases GPx activity in the biological systems. In particular, some of these studies [12], [35] have linked such increase with neuroprotective effects. However, most of the current literature on this theme is composed of either (i) phenomenological studies that only describe higher GPx activity [36] or (ii) studies that show or hypothesize that the observed higher GPx activity represents the consequence of the compound's thiol-peroxidase activity [35]. Here, we reported a mechanism for the neuroprotective effect of (PhSe)_2_ that goes far beyond its known GPx mimetic activity. Herein, we reported that (PhSe)_2_ was able to increase GPx by stimulating the mRNA expression of GPx1 (but not GPx4). Of note, experiments with mercaptosuccinic acid (GPx inhibitor) showed that the observed increase in GPx activity was necessary for the observed protection of (PhSe)_2_ against tert-BuOOH. In addition, (PhSe)_2_ also caused a significant increase in GSH levels, which were paralleled by an increase in *Gclc* expression. These results shed light into novel mechanisms concerning the antioxidant effects of this compound.

Oxidative stress and mitochondrial dysfunction have been reported as critical factors in the pathophysiology of neurodegenerative diseases [1], [37]. Although, the oxidants production is part of cellular homeostasis, their overproduction cause mitochondrial dysfunction that can culminate in energetic impairment and cell death [38], [39]. According to previous studies [40], [41], [42], here we observed an intense production of oxidants induced by tert-BuOOH exposure in HT22 cells from the first minutes of exposure (Fig. 3) that promoted mitochondria dysfunction culminating later in cell death. By contrast, the pre-incubation of cells with a nontoxic concentration of (PhSe)_2_ rendered cells more efficient in detoxifying tert-BuOOH (or derived oxidants), which in turn prevented the cell death, showing that these cells can cope better with pro-oxidant situations. This *in vitro* observation can better explain the neuroprotective effect of (PhSe)_2_ previously described in *in vivo* models of neurodegenerative diseases, such as Alzheimer disease (AD) [11] and neurotoxicity (CdCl_2_, H_2_O_2_, methimazole, and ischemia/reperfusion) [43], [44], [45], [46].

Mitochondria are also critical regulators of cell death [47], [48]. Many lines of evidence suggest that mitochondrial dysfunction occurs early and acts causally in neurodegenerative disease pathogenesis [1]. Therefore, protection of mitochondrial integrity and function is emerging as a promising strategy to prevent neuronal damage. As expected, the intense oxidants production induced by tert-BuOOH caused a fast and significant impact on mitochondrial function in HT22 cells. Decreases in the maximum OCR and mitochondrial reserve capacity were observed as early as 2 h of exposure to tert-BuOOH (40 µM) (Fig. 4B), therefore before the induction of cell death (which occur in 12 h – Fig. 1C). A slightly longer exposure to tert-BuOOH (4 h) further impaired the mitochondrial function by reducing the basal OCR, the ATP-linked oxygen consumption, the maximum OCR and mitochondrial reserve capacity (Fig. 4C). The excessive amount of oxidants produced by tert-BuOOH would leads to oxidation of mitochondrial proteins resulting in mitochondrial dysfunction, bioenergetic impairment and exponential production of oxidants. In fact, the decrease in mitochondrial reserve capacity has been described as a strong indicator of mitochondrial dysfunction [49]. Similarly, a loss of mitochondrial reserve capacity was observed in photoreceptor cells exposed to tert-BuOOH [50]. Interestingly, our results indicate that the pre-incubation with (PhSe)_2_ was efficient in preventing mitochondrial dysfunction induced by tert-BuOOH probably by neutralizing this peroxide or derived oxidants. In agreement with these data, previous studies from our group has demonstrated that (PhSe)_2_ improve the mitochondrial reserve capacity and therefore prevented oxidant-induced mitochondrial dysfunction in endothelial cells [15], [51]. Although, our results showed that (PhSe)_2_ protects HT22 cells from tert-BuOOH-induced mitochondrial dysfunction, we did not observed its ability in increase mitochondrial reserve capacity (Fig. 4). Together, these findings reinforce the idea that (PhSe)_2_ can maintain the mitochondrial function in oxidative stress conditions, thus preserving the bioenergetic and functional integrity of mitochondria in HT22 neuronal cells exposed to tert-BuOOH.

Recent studies postulate that the antioxidant property of (PhSe)_2_ goes far beyond its mimic action to the GPx [14], [51]. In fact, the protective action of (PhSe)_2_ was observed at a low concentration (2 µM) and time-dependent, discarding an exclusive scavenger effect linked to its peroxidatic activity. Additionally, it is important to consider that the simple thiol-peroxidase activity of (PhSe)_2_, is approximately 3–4 order lower than that of native GPxs ([18], seem to be not enough to justify its well-reported antioxidant properties in biological systems [17]. Based on our first results (Figs. 1–3), we hypothesized that the effect of (PhSe)_2_ in preserving mitochondrial function and cell survival could be due to the increase in cellular antioxidant defenses. Our results showed that (PhSe)_2_ positively modified the glutathione-dependent antioxidant system in HT22 cells by increasing the *Gpx1* gene expression and the GPx activity, as well as the level of its co-substrate GSH, through the increase in the expression of *Gclc* (a gene encoding γ-glutamylcysteine synthetase, which is the limiting enzyme in the GSH pathway) (Fig. 5). This fast and efficient cellular antioxidant modulation mediated by (PhSe)_2_ made these cells more resistant to oxidative damage. It is known that GSH is part of the glutathione peroxidase system, as an electron donor that reacts directly with free radicals [52] and its reduction has been associated with a loss of the brain antioxidant defense in neurodegenerative conditions [53]. In this context, compounds that may induce an increase or renewal of GSH levels are of great relevance in future therapies for neurodegenerative diseases.

Studies on the antioxidant effect of (PhSe)_2_ toward tert-butyl hydroperoxide are available in the literature. In fact, Ibrahim and colleagues [54], [55], [56] reported the antioxidant effects of some organoselenium compounds, including (PhSe)_2_ (used as a prototypal compound). The authors showed that although (PhSe)_2_ did not present DPPH radical scavenger activity, the organoselenium compounds were efficient in reducing *in vitro* oxidative stress markers in brain homogenates and attributed this effect to their GPx-like activity. It is important to mention that they evaluated the thiol-peroxidase activity in an exclusive chemical system. Therefore, the GPx-like activity was evaluated in absence of viable cells or more complex biological samples, which made impossible the occurrence of events such as transcription and/or translation. Those studies and others are relevant to determine the potency of organoselenium compounds regarding its chemical property to reduce peroxides. Moreover, we previously evaluated the kinetic reaction between (PhSe)_2_ and peroxynitrite in a stopped flow spectrophotometer and we found that they did not react at an appreciable rate (*k*_*2*_ < 10^4^ M^−1^ s^−1^) [14]. In addition, the direct oxidation of the selenium atom of organic selenides by peroxides seems to occur only when peroxides are present at the milimolar concentrations [57], which have no biological relevance under our experimental conditions. The results of the present study (especially Fig. 6, Fig. 7) strongly suggest that we evaluated the enzyme activity (with no interference of the compound's GPx-like activity), considering the low concentrations of (PhSe)_2_ used in our experiments (low micromolar). The increased levels of GPx1 mRNA levels reinforce this idea.

To evaluate the contribution of GPx activity on the cytoprotective effect of (PhSe)_2_, we exposed (PhSe)_2_-pre-incubated HT22 cells to MS, an inhibitor of the GPx enzyme, slightly before tert-BuOOH exposure. Our results showed that the MS decrease the effect of (PhSe)_2_ in protecting HT22 cells against cell death induced by exposure to different concentrations of tert-BuOOH. Therefore, our results indicate that the positive modulation of *Gpx1* expression would be a key component for the protection afforded by (PhSe)_2_ in HT22 cells. It should be mentioned the importance of GPx activity, since its decrease promote susceptibility to oxidative stress by allowing the accumulation of harmful oxidants [58], [59], and because other selenoproteins cannot replace its function in protecting from generalized oxidative stress [5]. *Gpx4* gene expression was not upregulated after (PhSe)_2_ incubation and the results of decrease observed in the *mtGpx4* expression would not represent a biological significance event according to functional thresholds used in quantitative PCR analyses [60]. We can suppose that *Gpx1* isoform would be preferentially synthetized in (PhSe)_2_-treated HT22 cells, suggesting an interplay between the different isoforms and also suggest that (PhSe)_2_ regulates *Gpx1*-specific transcriptional machinery in HT22 cells that does not involve *Gpx4*. Contrary to our results, (PhSe)_2_ did not increase the GPx activity and protein expression in neuroblastoma cells [61], indicating that these cells would present a different physiological response than those found in HT22 cells. Together these evidences reinforce the idea that this simple organoselenium compound acts as an indirect and effective antioxidant by modulating intracellular redox-sensitive responses.

Our results disclose that the molecular mechanism of neuroprotective actions of (PhSe)_2_ involves, at least in part, the GPx1 modulation, however we can not discard the contribution of other antioxidant enzymes in its effects. In an *in vivo* study, our group showed that (PhSe)_2_ protected against methylmercury (MeHg)-induced mitochondrial changes in the cerebral cortex of mice [62]. In the same study, we observed that (PhSe)_2_ increased HO-1 content in cultured astroglial cells (C6 lineage). In that study, the achieved results (mainly those related to HO-1 expression) leaded us to hypothesize that (PhSe)_2_ would trigger the cytoprotective Nrf-2 pathway, although no evidence-based results were provided [62]. According, some lines of evidence propose that the “thiol modifier effects” of organoselenium compounds might be more relevant for the explanation of their pharmacological effects than their GPx-like activity [17]. Based on the fact that diselenides are weak electrophiles, it is suggested that they can oxidize critical cysteinyl residues in Keap1, allowing Nrf-2 to transcriptionally activate the expression of antioxidant enzymes [14], [17], such as HO-1, which is a sensitive marker of the activation of Keap1/Nrf-2 signaling pathway. In order to delve into this topic, here we evaluated some Nrf-2-target genes in HT22 cells after (PhSe)_2_ treatment. The results (Fig. 7) showed an early increase in *HO-1* gene expression promoted by (PhSe)_2_ treatment. Notably, additional Nrf-2-target genes were also positively modulated by (PhSe)_2_, such as Gclc (encoding the rate-limiting enzyme in glutathione biosynthesis) and Cat (long-lasting effect). According, we previously showed that (PhSe)_2_ induced redox regulation and nuclear localization of Nrf-2 in endothelial cells [14]. Some evidence suggests that GPx1 transcription may be regulated directly or indirectly by Nrf-2. The expression of GPx1 was downregulated in lung after exposure to cigarette smoke in Nrf-2-knockout mice [63], while, the enhanced Nrf-2 expression increased GPx1 transcription and decreased oxidants generation in glioma stem cells [64]. These results reinforce the idea that (PhSe)_2_ triggers the cytoprotective Nrf-2 pathway.

Similarly to Nrf-2, FoxO-3 (forkhead box, class O-3) is also a redox-regulated transcription factor involved in the control of stress-mediated cellular responses. Of note, there is emerging evidence to the link between FoxO/Nrf-2 activation [65]. Some studies also support the participation of FoxO-3 in GPx1 modulation. In chondrocytes, the constitutive active form of FoxO-3 induced GPx1 while increasing cell viability in response to tert-BuOOH [66]. Beside, the expression of GPx1 was downregulated in FoxO-3-deficient erythroid precursor cells [67]. Herein, the genic expression of some FoxO-3-regulated antioxidant enzymes were analyzed. Only *Gpx1* and *Cat*, which would be trigged by either Nrf-2 or FoxO-3, were upregulated by (PhSe)_2_, while *Sod2, Txnrd2, Prdx3* and *Prdx5*, classically regulated by FoxO-3, were not modulated by this organoselenium compound. We have previously shown that (PhSe)_2_ increased the expression of Prdx3 and Sod2 [15] and improved mitochondrial function in endothelial cells [51], suggesting the involvement of FoxO-3 activation. In this sense, the contribution of FoxO-3 pathway in the protective actions of (PhSe)_2_ in HT22 cells should not be completely ruled out. Therefore, it is likely that the Nrf-2 and/or FoxO-dependent signaling cascade(s) would be trigged by (PhSe)_2_ through its “thiol modifier effects”, and these responses would depend of the cellular type.

In conclusion, we propose in the graphical abstract some molecular mechanisms activated by (PhSe)_2_ in HT22 cells that allow the protection against the oxidative stress induced by tert-BuOOH. Our results demonstrated that (PhSe)_2_ orchestrated an adaptive cellular response to oxidative stress through *Gpx1* modulation, which allowed cells to neutralize oxidants and, as consequence, prevent mitochondrial dysfunction and cell death. This study shows that the cytoprotective effects of (PhSe)_2_ goes far beyond its well-known thiol-peroxidase activity. In contrast with the findings obtained in simple chemical systems, where organoselenium compounds exhibits thiol-peroxidase-like activity, in the complex scenario of living neuronal cells, (PhSe)_2_ has indirect GPx-like activity. Importantly, in view of the superior efficiency of native GPx enzymes over simple organoselenium compounds, the thiol modifiers properties of (PhSe)_2_ and analogs have to be exploited in the searching for effective antioxidant therapeutic agents.
